# Non-Iterative Optimal Design Method Based on LM Index for Steel Double-Beam Floor Systems Reinforced with Concrete Panels

**DOI:** 10.3390/ma15134538

**Published:** 2022-06-28

**Authors:** Insub Choi, Dongwon Kim, JunHee Kim

**Affiliations:** Department of Architectural and Architectural Engineering, Yonsei University, Seoul 03722, Korea; insub@yonsei.ac.kr (I.C.); kdw6797@yonsei.ac.kr (D.K.)

**Keywords:** non-iterative optimal design, LM index, steel double-beam floor system, rotational constraints, greenhouse gases

## Abstract

Steel double-beam floor systems reinforced with concrete panels can improve the structural and environmental performance of buildings by reducing moment demands and embodied CO_2_ emissions. However, for steel double-beam floor systems, a time-consuming iterative analysis is required to derive an optimal design proposal owing to the rotational constraints in the composite joints between the concrete panel and steel beams. In this study, a non-iterative optimal design method using the LM index is proposed to minimize the embodied CO_2_ emissions of steel double-beam floor systems. The LM index is a measure that can be used to select the optimal cross-section of the steel beams considering the decreased moment capacity according to the unbraced length. The structural feasibility of the proposed design method was verified by investigating whether safety-related constraints were satisfied by the LM index with respect to the design variables under various gravity loads. The applicability of the proposed optimal design method is verified by comparing the embodied CO_2_ emissions derived from the proposed and code-based design methods. Applicable design conditions were presented based on the LM index to aid engineers. The proposed design method can provide environmentally-optimized design proposals to ensure structural safety by directly selecting the LM index of steel beams.

## 1. Introduction

As urbanization has accelerated to address population growth, construction methods are required to efficiently utilize the limited space available in downtown areas. Top-down methods [1,2] are alternatives for constructing new buildings in downtown areas by securing underground spaces, and steel floor systems which are widely used in underground structural systems to increase workability [3]. Apart from improving the insulation performance of buildings [4,5,6,7], a reduction in the production of materials such as cement and steel which generate 11% of the total greenhouse gases (GHGs) [8] can significantly contribute to solving global warming problems. It is well known that replacing cement with fly ash reduces GHG emissions [9], but concrete buildings have an inherent problem of emitting significant GHG during the service life of buildings including repair and maintenance [10]. In this regard, steel–concrete composite floor systems capable of reducing material quantities are preferred for improving the environmental performance of buildings [11].

The steel–concrete composite floor system is more advantageous than steel floor systems for reducing material quantities and GHGs related to embodied CO_2_ emissions because it can increase the stiffness and strength of the section owing to the composite effect of steel and concrete [12]. Kinderis et al. [13] reported that the composite floor system, the so-called Deltabeam, can reduce the building’s height compared to concrete beams. Du et al. [14] experimentally confirmed that high-strength materials can dramatically improve the flexural performance of the steel–concrete composite floor system. In addition, the flexural performance of composite floor systems can be improved by increasing the degree of composite between steel and concrete [15], but it is difficult to construct in-situ due to complex details. Although the constructability of the steel–concrete composite floor systems can be improved through bolted connections between the beam and column, these systems are disadvantageous in reducing material quantities owing to the increased moment demand at beam members under high gravity loads. To improve rotational constraints on composite connection, Ju et al. [16] proposed a TEC (technical, economical, and convenient) beam having code conforming rigid connections. Amadio et al. [17] showed that steel truss embodied connection effectively increases the flexural performance of the connection. Other studies [18,19] have been conducted to increase the rotational constraints of beam–column connections to save the materials. However, complicated details are still required to secure sufficient rotational constraints at the connections; therefore, there is a disadvantage in that the constructability of these steel–concrete composite floor systems decreases.

Recently, a steel double-beam floor system [20] was developed to reduce material quantities and improve constructability compared with a general beam–girder (GBG) system. The steel double-beam floor system reinforced with concrete panels at the double-beam ends is an eco-friendly floor system that can significantly reduce embodied CO_2_ emissions related to material quantity, even under high gravity loads [21]. By applying optimal design methods to steel double-beam floor systems, additional reductions in the embodied CO_2_ emissions on manufacturing structural members are possible. Szewczyk and Szumigała [22] derived an optimal design proposal that can minimize the cost-related production of materials for a steel–concrete composite beam. Optimal design methods generally require time consuming iterative analyses to minimize objective functions [23,24,25]. In the case of a multi-objective problem [26,27,28,29] considering cost and environmental impacts, a more iterative analysis is required to derive an optimal design proposal that minimizes the objective function while satisfying structural safety. A genetic algorithm (GA) is a good tool to solve the multi-objective problem [30], and the analysis time can be greatly reduced through the improvement of the algorithm [31]. However, when the composition of the steel–concrete beam is changed, it is necessary to calibrate the structural or design parameters constituting the GA. Moreover, because the rotational constraints of composite connections needed to be evaluated using sophisticated numerical models [32,33,34,35] or hybrid models [36,37] to calculate the member forces, the computational cost is significantly increased to derive the optimal design proposal for a steel double-beam floor system reinforced with concrete panels. Data intensive approaches such as back-propagation neural network (BPNN) [38] and particle swarm optimization (PSO) [39] can be used as alternatives. However, because these methods require an extensive database from iterative analysis, they are difficult to utilize in the practical field. Therefore, to design a steel double-beam floor system reinforced with concrete panels, considering the rotational constraints of composite connections in the practical field, a new optimal design method should be developed such that practical engineers can use it without time consuming iterative analyses.

In this study, an efficient optimal design method for steel double-beam floor systems was developed by simply providing the design parameters without iterative analysis. By introducing a new index called the LM index on the design parameters and formulating the objective function, the optimal cross-section of the steel beams can be selected by minimizing the material quantity related to the embodied CO_2_ emissions. The design feasibility of the developed optimal method is verified by comparing it with the material quantity calculated using a code-based design method. Finally, the applicable conditions for designing steel double-beam floor systems are presented based on the LM index with a histogram of the limiting laterally unbraced length for H-beams available in practice.

## 2. Non-Iterative Optimal Design Method Using LM Index

### 2.1. Description of Steel Double-Beam Floor Systems

**Figure 1:**
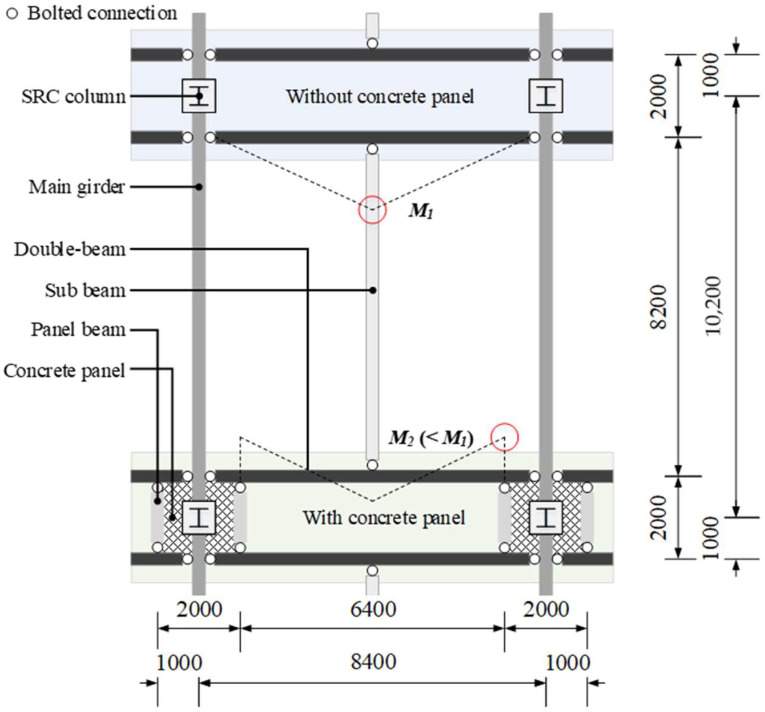
Structural plan of steel double-beam floor systems and comparison of bending moment of double-beam according to the absence of concrete panel (unit in mm).

As shown in Figure 1, the steel double-beam floor system consists of steel-reinforced concrete (SRC) columns, steel beams, concrete panels, and steel double-beams installed in a short direction to distribute gravity loads. Bolted connections were used to improve the in-situ workability of the steel double-beam floor system for the connections between the double-beam and the main girder corresponding to the beam–column connection of the GBG system. Under high gravity loads with live loads exceeding 6.0 kN/m^2^, the material quantity of the steel double-beam system without a concrete panel (DBX) increased owing to the increase in the moment demand of the double-beam (i.e., *M*_1_ indicated as red-circled in Figure 1); therefore, the material quantity of the DBX system would be increased compared to the GBG system [21]. A steel double-beam floor system reinforced with a concrete panel (DBO) can reduce the moment demand of the double-beam (i.e., *M*_2_ indicated as red-circled in Figure 1) by installing a beam end concrete panel that reduces the effective length of the double-beam and induces negative moments at the beam end.

The rotational constraint induced by the concrete panel was quantitatively evaluated by considering the practical boundary conditions from a previous study [20]. The stiffness ratio (*μ*), defined as the ratio of the rotational stiffness of the composite connection to the flexural stiffness of the double-beam, was determined to be 0.032, which represents a code conforming to the rigid connection suggested by ANSI/AISC 360-16 (American National Standards Institute/American Institute of Steel Construction) [40]. From Kirchhoff–Love plate theory, the design parameter of the concrete panel (i.e., thickness) can be calculated using Equation (1) to secure a rotational constraint equivalent to a code conforming rigid connection. Because the design parameters of the double-beam (i.e., the moment of inertia) and concrete panel (i.e., thickness) are dependent, a time consuming iterative analysis is required to optimize the material quantity of the DBO system.

(1) $$T_{P}=\sqrt[{3}]{\frac{12\left(1-v^{2}\right)}{E_{p}}\frac{\left(EI/L\right)}{\mu }}$$

where *T_p_* is the thickness of the concrete panel (mm), *E_p_* is the elastic modulus of the concrete panel (24,422 MPa; calculated according to ACI 318-19 [41] for compressive strength of 27 MPa), *E* is the elastic modulus of the double-beam (205,000 MPa), *I* is the moment of inertia of the double-beam (m^4^), *L* is the length of the double-beam (m), *μ* is the stiffness ratio, and *v* is Poisson’s ratio of concrete (0.15).

### 2.2. Formulation of Objective Function Using LM Index

**Figure 2:**
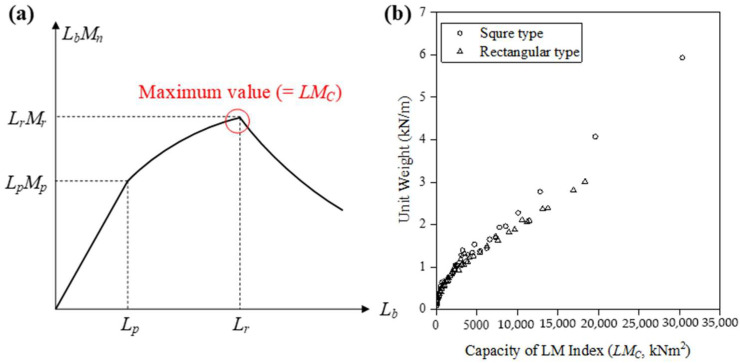
Description of the LM index in hot-rolled H-beam. (**a**) Relationship with the unbraced length. (**b**) Unit weight according to the capacity of the LM index.

In general, the cross-sectional areas of the structural members are used as the objective function to minimize the material quantity in steel frames [42]. To solve the dependency of the design parameters between the double-beam and concrete panel, in this study, the LM index (*L_b_M_n_*) has been defined by multiplying the unbraced length (*L_b_*) and the nominal flexural strength (*M_n_*) of steel beams. As shown in Figure 2a, the LM index has a maximum value in *L_r_*; therefore, the LM index in terms of capacity (*LM_C_*) is defined as Equation (2) using this unique characteristic. Similarly, the LM index in terms of demand (*LM_D_*) was defined by Equation (3) from the results of the structural analysis.

(2) $$L_{b}^{i}M_{n}^{i}\leq L_{r}^{i}M_{r}^{i}=LM_{C}^{i}$$

(3) $$L_{b}^{i}M_{u}^{i}=LM_{D}^{i}$$

where *L^i^_b_* is the unbraced length of the steel beams (m), *L^i^_r_* is the limiting laterally unbraced length for the limit state of inelastic lateral-torsional buckling (m), *M^i^_r_* is the inelastic bending moment for lateral-torsional buckling (kNm), *LM^i^_C_* is the LM index in terms of capacity (kNm^2^), and *LM^i^_D_* is the LM index in terms of demand (kNm^2^). The superscript *i* denotes *i*-th steel member.

Figure 2b shows the unit weight for practically available 95 H-beams suggested in KS D 3502 [43] in accordance with the *LM_C_*. Because the unit weight and the *LM_C_* was proportional, the values of the *LM_C_* can be considered as a weight. Therefore, the objective function for minimizing the material quantity can be established in Equation (4) in terms of the LM index as follows:

(4) $$Minimize\;W_{LM}=\sum_{i=1}^{M}\left\{\phi_{b}LM_{C}^{i}-LM_{D}^{i}\right\}$$

where *W_LM_* is the objective function of the material quantity expressed in the LM index (kNm^2^), *ϕ_b_* is the flexural strength reduction factor (0.9 suggested from AISC 360-16 [40]), and the superscript *i* denotes the *i*-th steel beam.

### 2.3. Constraint Conditions Conforming Design Codes

To Conform to current design codes, such as ASCE 7-16 [44] and ANSI/AISC 360-16 [40], safety-related constraint conditions are employed, as shown in Equations (5) and (6) based on the deflection and strength conditions of the steel beams, respectively. Under the service load defined as 1.0 D.L (dead load) + 1.6 L.L (live load), the deflection of the steel beams shall not exceed 1/480 of the length of the steel beam. Also, under the factor defined as 1.2 D.L + 1.6 L.L, the nominal flexural strength of steel beams considering strength reduction along the unbraced length should be greater than the moment demand (i.e., maximum moment).

(5) $$\frac{\delta_{L}^{i}}{L_{s}^{i}/480}\leq 1.0$$

(6) $$\frac{M_{u}^{i}}{\phi_{b}M_{n}^{i}}\leq 1.0$$

where *δ^i^_L_* is the maximum deflection of the *i*-th steel member under the service load, *L^i^_s_* is the length of the *i*-th steel beam, *M^i^_u_* is the moment demand of the *i*-th steel beam (kNm), *ϕ_b_* is the flexural strength reduction factor (0.9), and *M^i^_n_* is the nominal flexural strength of the *i*-th steel beam determined by considering the strength reduction according to the unbraced length.

Although the code conforming constraint conditions can be considered using Equations (5) and (6), iterative analysis is still required in the optimal design process to minimize the objective function defined by Equation (4) because the objective function is not defined with respect to the LM index. To organize the constraint conditions into the LM index, the denominator and numerator on the left-hand side of Equation (6) were multiplied by the unbraced length of each member (*L^i^_b_*). Then, from the definition of the LM indices in terms of demand and capacity summarized in Equations (2) and (3), the constraint condition for the strength can be expressed for the LM index as shown in Equation (7). Additionally, Equation (8) is employed as a strength-related constraint condition to prevent the violation of the design code.

(7) $$\frac{LM_{D}^{i}}{\phi_{b}LM_{C}^{i}}\leq 1.0$$

(8) $$\frac{M_{u}^{i}}{\phi_{b}M_{p}^{i}}\leq 1.0$$

where *M^i^_p_* is the plastic bending moment (kNm).

### 2.4. Non-Iterative Optimal Design Process

**Figure 3:**
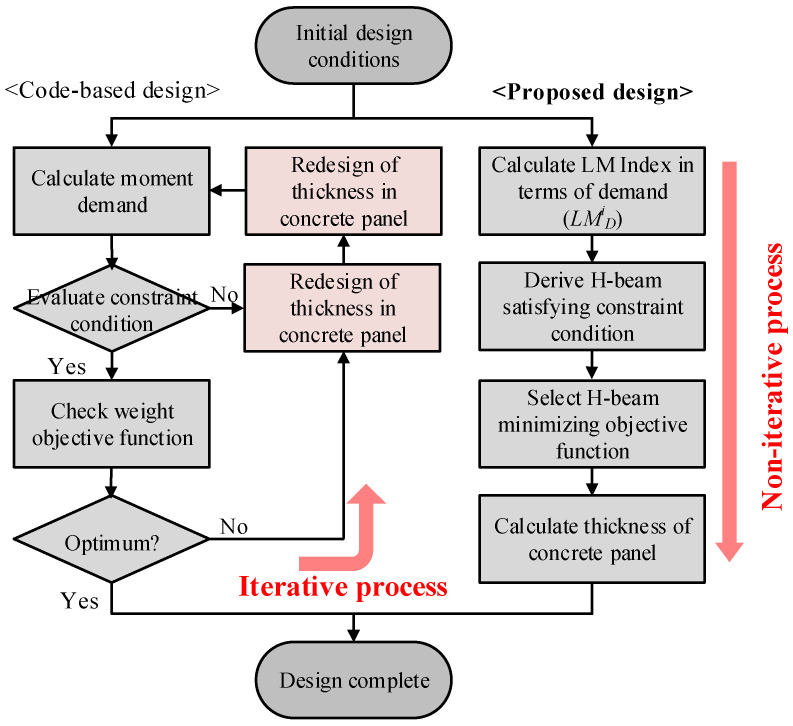
Comparison of optimization processes between code-based design and proposed optimal design methods.

Figure 3 shows a comparison of the optimization process between the code-based and proposed optimal design methods. In the code-based design method, the iterative analysis is required to minimize the material quantity because the redesign of the steel beams leads to the redesign of the concrete panel. However, the proposed optimal design method does not require iterative analysis for quantity optimization as shown in Figure 3. By comparing the objective functions under the constraint condition, it can be seen that the constraint condition is automatically satisfied by minimizing the objective function. Therefore, by introducing the LM index as the design parameter in the developed optimal design method, the optimal design proposal minimizing the material quantity can be derived by simply selecting the cross-section of the steel beams with the smallest *LM_C_* exceeding the *LM_D_* without repeated structural analysis. Subsequently, the thickness of the concrete panel, which can induce the rotational constraint corresponding to the rigid connection, is calculated using Equation (1).

## 3. Validation of Proposed Optimal Design Method

### 3.1. Typical Design Conditions of Underground Structures Used for Parking Lots

**Figure 4:**
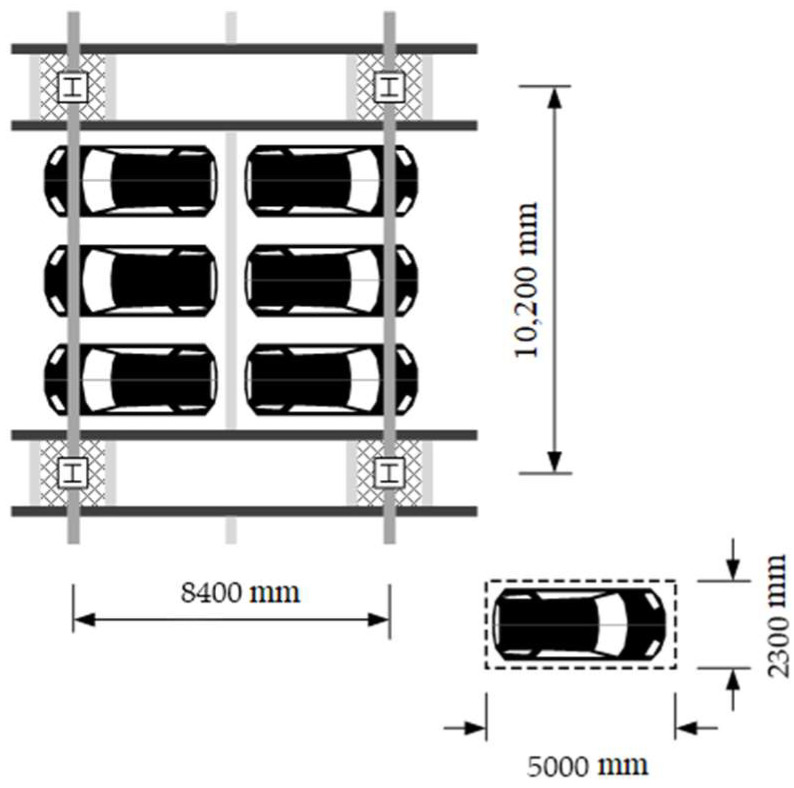
A representative floor plan of underground structures considering the column spacing for parking lots.

Figure 4 shows a typical floor plan for the underground structure of steel buildings used as parking lots. According to the Korean Design Standard (KDS) [45], the unit parking space for one vehicle must be at least 2.3 m × 5.0 m. Based on the unit parking space, the typical dimension of the frame was determined to be 8.4 m × 10.2 m for accommodating the parking space of six vehicles. To investigate the feasibility of the developed optimal design method using the LM index, underground structures having five spans in the short side direction (i.e., 8.4 m) and three spans in the long side direction (i.e., 10.2 m) were considered (see Section 3.2 for detailed information).

Since the moment demands of the underground structures were determined by the gravity load, the live loads according to the usage of the underground space were divided into five categories as summarized in Table 1 based on ASCE 7-16 [44]. Note that the live load of the underground space of the knowledge industry center is practically designed to be 8.0 kN/m^2^ in Korea. The dead load was set to be 5.2 kN/m^2^ including the thickness of the concrete slab of 150 mm and finishing materials from previous work [21]. SM490 with a yield strength of 325 MPa was used for the steel to calculate the capacity of the steel beams and the compressive strength of the concrete was set to 27 MPa. By considering various gravity loads, it is possible to examine whether the developed optimal design method can be used in practical applications and to suggest feasible design conditions under realistic constraints.

### 3.2. Structural Modeling for Steel Double-Beam Floor Systems

**Figure 5:**
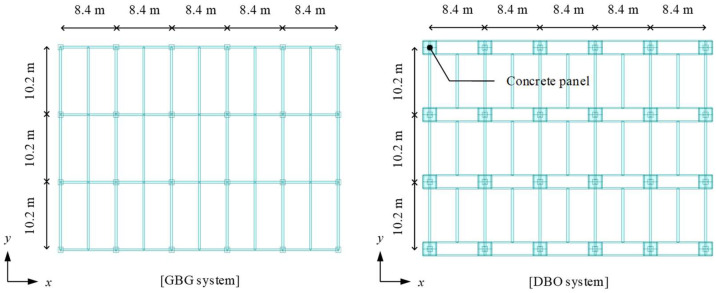
Illustration of standard frame models of the typical underground structures applied to three different floor systems (Note that the DBX system has the same configurations as the DBO system except for the concrete panel).

As shown in Figure 5, standard frame models of the typical underground structures are considered to calculate the moment demands for five live loads. MIDAS-Gen [46] was used for frame analysis and the dimensions of the structural plan are summarized in Figure 5. If the cross-section of the H-beam initially assumed in a code-based design does not satisfy safety-related constraints, the cross-section of the H-beam should be changed because safety-related constraint conditions are mandatory in the structural design. On the other hand, in the proposed design method, the cross-section of the H-beam can be directly selected using the *LM_C_*, which minimizes the difference from the *LM_D_*. A comparison of the optimal design proposal between the code-based and proposed optimal design methods is presented in Section 3.3.

**Figure 6:**
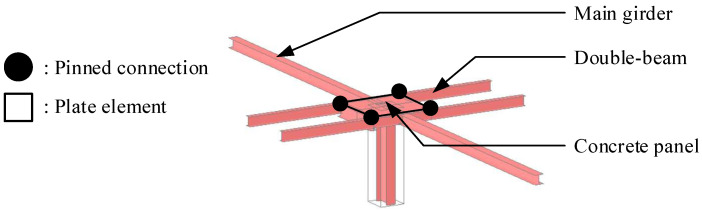
Modeling of connections using plate elements with beam-end release conditions in the steel double-beam floor system reinforced with the concrete panel (DBO system).

The connection between the column, girder, and main beam was modeled using a rigid connection in the GBG system. All connections in the DBX and DBO systems except for the connection between the main girders and columns were modeled as an ideal pinned connection using the built-in option beam end released condition in the MIDAS-Gen. To consider the rotational constraints induced by the concrete panel in the DBO system, as shown in Figure 6, a concrete panel with a width of 2.0 m and a thickness of 0.04 m was modeled as a plate element. The elastic modulus of the concrete panel was set to 24,422 MPa based on the compressive strength of the concrete. For the five gravity load cases including the live loads summarized in Table 1, a series of frame analyses were performed to calculate the moment demand of each floor system for a typical floor.

### 3.3. Design Feasibility of Proposed Optimal Design Method

The design feasibility of the proposed method was examined for the five gravity load cases. The moment demand and *LM_D_* for the main beam or double-beam are summarized in Table 2. The model name contains the applied floor system and live load condition; for example, DBOL2 indicates a model with live load condition L2 (i.e., 4 kN/m^2^) applied to the DBO system. While the moment demands for the main beam in the GBG system and the double-beam in the DBX system have similar values, the moment demand for the double-beam in the DBO system decreases by 63% compared with that in the DBX system owing to the beam-end constraints of the concrete panel. In other words, the installation of the concrete panel at the ends of the double-beam can lead to a reduction in the material quantity of steel double-beam floor systems.

The optimal design proposal for DBOL4 and DBOL5 under high gravity loads exceeding the live loads of 6.0 kN/m^2^ derived by the code-based and proposed methods were summarized in Table 3 based on the results of the frame analysis. All structural members designed using both methods had the same cross-section of the H-beam. The optimal design proposal using the proposed method can be derived without repeated analysis by easily selecting the cross-section of the H-beam with an *LM_C_* that minimizes the difference from an *LM_D_*. Therefore, the developed optimal design method has a high potential to be utilized in the practical field while avoiding time consuming repetitive analysis. To further examine the feasibility of the proposed design method, the material quantities designed by the two methods were compared for each floor system under five live loads.

**Figure 7:**
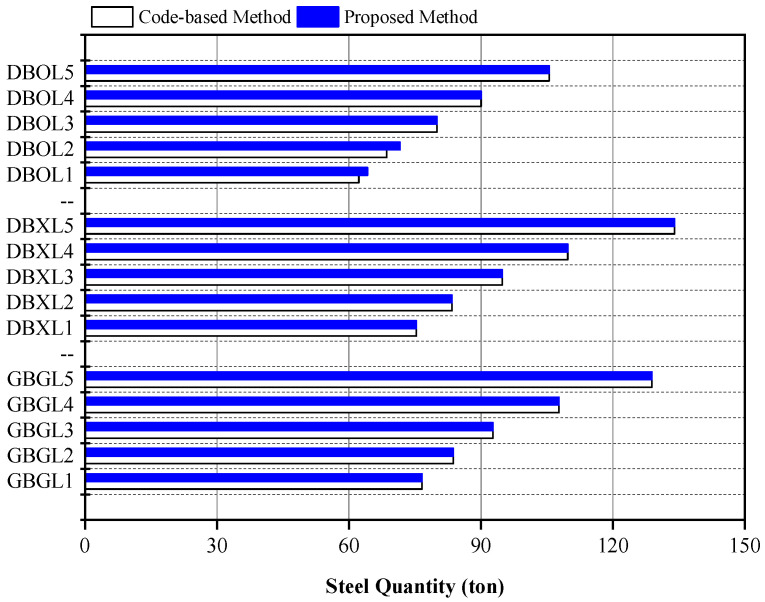
Designed steel quantity for each floor system from code-based and proposed methods.

The quantities of steel for each floor system derived using the code-based and proposed methods are summarized in Figure 7. The quantities of steel derived by the two design methods were similar for the GBG and DBX systems regardless of the live loads. For the DBO system, the design proposals derived using the two design methods were equal when the live loads were greater than 6.0 kN/m^2^. This result indicates that the proposed optimal design method for the DBO system provides an optimal design proposal equivalent to the code-based method under high gravity loads exceeding the live loads of 6.0 kN/m^2^. Therefore, the proposed optimal design method using the LM index is applicable for three different floor systems by providing a quantity-optimized design output similar to the code-based design method without time consuming iterative analysis.

### 3.4. Environmental Efficiency of Rotational Constraints

The increment of the embodied CO_2_ emissions owing to the addition of the concrete panel has little impact on the total embodied CO_2_ emissions of structural materials. However, the rotational constraint induced by the concrete panel can significantly improve the environmental performance of the DBO system compared with the GBG system by reducing the embodied CO_2_ emissions related to steel quantities. To evaluate the environmental efficiency of the rotational constraint induced by the concrete panel, the global warming potential (GWP) was calculated using Equation (9) [47] from the steel quantity derived using the proposed optimal design method. For the DBX and GBG systems, the embodied CO_2_ emissions required for the calculation of the GWP were determined using Equations (10) and (11) [47] from the steel quantity summarized in Figure 7. For the DBO system, the embodied CO_2_ emissions were calculated by considering the steel quantity in addition to the concrete quantity for the concrete panel.

(9) $$\mathrm{GWP}={\mathrm{CO}}_{2,Embodied}\times \mathrm{CF}$$

(10) $${\mathrm{CO}}_{2,Embodied}=\sum_{j}{\mathrm{CO}}_{2,Embodied,j}$$

(11) $${\mathrm{CO}}_{2,Embodied,j}=\rho_{j}m_{j}$$

where CF is the characterization factor for the GWP (1.00 kgCO_2_-eq/kg from IPCC 2013 [48]), CO_2,*Embodied*_ represents the embodied CO_2_ emission of whole building materials, CO_2,*Embodied*,*j*_ represents the embodied CO_2_ emission of the *j*-th building material, *ρ_j_* is the carbon equivalent emission value for the *j*-th building material (kgCO_2_-eq/kg), and *m_j_* is the mass of the *j*-th building material (kg). Note that *ρ* values for the concrete material and the steel material were used as 0.141 kgCO_2_-eq/kg and 1.42 kgCO_2_-eq/kg, respectively, based on ICE-DATA v2.0 [49,50].

**Figure 8:**
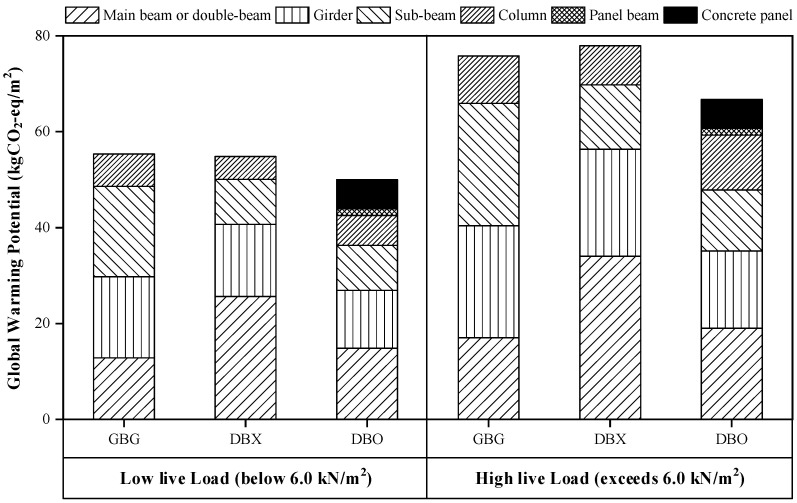
Comparison of global warming potentials (GWPs) between the general beam-girder system (GBG) and the double-beam floor systems with concrete panel (DBO) and without concrete panel (DBX).

Figure 8 shows the GWPs between the DBX and DBO systems in accordance with the presence of the concrete panel with the GBG system. For low live loads (below 6.0 kN/m^2^), the DBX system had a slightly smaller GWP than the GBG system, but the DBO system shows a smaller GWP value compared to the DBX system. In particular, for high live loads (exceeding 6.0 kN/m^2^), while the DBX system has a higher GWP value than the GBG system, the DBO system has a smaller GWP value than the GBG system. Therefore, the installation of concrete panels improves the environmental performance of steel double-beam floor systems more effectively under high gravity loads. Furthermore, the proposed optimal design method can be practically utilized in quantity optimization processes that require environmental performance evaluation. Since life cycle assessment (LCA) is becoming important in the construction sector related to buildings and bridges [51], the proposed method could be used in the LCA by evaluating costs comprised of material production and embodied CO_2_ emissions in future studies.

### 3.5. Applicable Design Conditions Using LM Index

As the results of quantity optimization for the three different floor systems applied to the underground structures used for parking lots, the design feasibility of the proposed method was validated through the comparison of the steel quantity of beam members derived from the code-based design method. When *L_r_* and *L_b_* are the same, the designed cross-section of the H-beams based on the LM index satisfies both the safety-related constraint conditions and the objective function of minimizing the steel quantity. Because the ranges of *L_r_* used in the calculation of *LM_C_* and *L_b_* used in the calculation of *LM_D_* were similar in the designed cross-section of the H-beams, the proposed design method could yield reliable optimal design proposals regardless of the applied floor systems.

**Figure 9:**
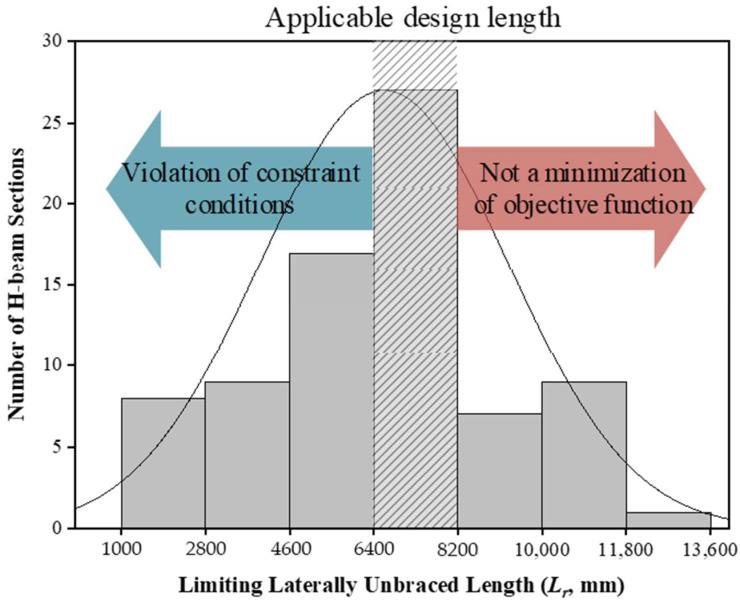
Applicable design length of steel beams using the LM index with histogram according to the limiting laterally unbraced length of the H-beams available in practice.

For the designed cross-section of the H-beams, the *L_r_* ranged from 5.2 m to 7.9 m. By considering the typical dimensions of the parking lots, the range of the *L_b_* was determined from 6.4 m to 8.2 m. Figure 9 shows the histogram of the number of the H-beams according to the *L_r_* to suggest the applicable design length using the LM index. The *L_r_* of the H-beams generally has a smaller value as the cross-sectional area or the moment of inertia is smaller. In a case where *L_r_* is less than 6.4 m (i.e., the lower bound of *L_b_*), there is a high possibility that the safety related constraint conditions will be violated because of a lower nominal flexural strength than the moment demand. However, when *L_r_* exceeds 8.2 m (i.e., the upper bound of *L_b_*), it is difficult to minimize the objective function because the nominal flexural strength of the H-beam significantly exceeds the moment demand. Therefore, in this study, since the range of the *L_b_* was determined from 6.4 m to 8.2 m by considering the typical dimensions of the parking lots, the applicable design length of the H-beams using the LM index can be suggested as 6.4–8.2 m indicated in shaded range of Figure 9.

## 4. Conclusions

An efficient optimal design method for steel double-beam floor systems was developed by simply providing design parameters without iterative analysis. For the planned parking lots of underground structures, the design feasibility of the developed optimal method was verified by comparing it with the material quantity calculated by the code-based design method. The following conclusions can be drawn from the findings of this study.

- By introducing a new index named the LM index on the design parameters and formulating the objective function, the optimal cross-section of the steel beams can be selected by minimizing the material quantity related to the embodied CO_2_ emissions.
- As a result, considering five categories of live loads ranging from 2.5 to 12.0 kN/m^2^, the proposed optimal design method was superb at providing a quantity optimized design option under the high gravity loads with a live load of 6.0 kN/m^2^ or more.
- The structural rotational constraint induced by the concrete panel can improve the environmental performance of the steel double-beam floor systems by reducing the GWP compared to the steel beam–girder floor system.
- The applicable design length of steel beams for practical engineers to use the proposed optimal method is suggested from 6.4 m to 8.2 m based on the LM index and plan of the typical underground structure used for parking lots.

## Figures and Tables

**Table 1:** Live loads according to the usages of the underground space [44].

Abbreviation	Usages	Live Load (kN/m^2^)
L1	Offices	2.50
L2	Passenger vehicles only	4.00
L3	Storage warehouses	6.00
L4	Knowledge industry center	8.00
L5	Heavy vehicles	12.00

**Table 2:** Results of moment demands and LM index in terms of demand for the main beam or double-beam from frame analysis.

Live Load (kN/m^2^)	Model	Mu (kNm)	LMD (kNm^2^)
2.5	GBGL1	489.5	2055.9
	DBXL1	482.5	2026.5
	DBOL1	181.2	579.8
4	GBGL2	600.5	2522.1
	DBXL2	594.3	2496.1
	DBOL2	222.8	713.0
6	GBGL3	747.8	3140.8
	DBXL3	740.7	3110.9
	DBOL3	278.6	891.5
8	GBGL4	896.5	3765.3
	DBXL4	891.3	3743.5
	DBOL4	336.2	1075.8
12	GBGL5	1196.4	5024.9
	DBXL5	1188.7	4992.5
	DBOL5	447.0	1430.4

**Table 3:** Optimal design proposal derived from code-based and proposed methods for the steel double-beam floor systems.

Model	Structural Member	Design Proposal	
		Code-Based Method	Proposed Method
DBOL4	Double-beam	H-400 × 200 × 8 × 13	H-400 × 200 × 8 × 13
	Girder	H-482 × 300 × 11 × 15	H-482 × 300 × 11 × 15
	Sub-beam	H-394 × 398 × 11 × 18	H-394 × 398 × 11 × 18
	Concrete Panel	*T_p_* = 0.250 m	*T_p_* = 0.250 m
DBOL5	Double-beam	H-386 × 299 × 9 × 14	H-386 × 299 × 9 × 14
	Girder	H-582 × 300 × 12 × 17	H-582 × 300 × 12 × 17
	Sub-beam	H-594 × 302 × 14 × 23	H-594 × 302 × 14 × 23
	Concrete Panel	*T_p_* = 0.320 m	*T_p_* = 0.320 m

## Data Availability

The data presented in this study are available on request from the corresponding author.
